# Do CYP2D6 genotypes affect oxycodone dose, pharmacokinetics, pain, and adverse effects in cancer?

**DOI:** 10.1080/14622416.2024.2430161

**Published:** 2024-12-04

**Authors:** Aaron K. Wong, Sara Vogrin, Pal Klepstad, Justin Rubio, Brian Le, Jennifer Philip, Andrew A. Somogyi

**Affiliations:** a Department of Palliative Care, Peter MacCallum Cancer Centre, Melbourne, Victoria, Australia; b Department of Palliative Care, The Royal Melbourne Hospital, Parkville, Victoria, Australia; c Department of Medicine, University of Melbourne Eastern Hill Campus, Fitzroy, Victoria, Australia; d Department of Medicine, St Vincent’s Hospital Melbourne, University of Melbourne, Victoria, Australia; e Department Intensive Care Medicine, St. Olavs University Hospital, Trondheim, Norway; f Principal Research Fellow, Florey Institute of Neuroscience & Mental Health, Victoria, Australia; g St Vincent’s Hospital, Palliative Care Service, Fitzroy, Victoria, Australia; h Clinical and Experimental Pharmacology, Discipline of Pharmacology, Faculty of Health and Medical Sciences, University of Adelaide, Adelaide, Australia

**Keywords:** Opioid, pharmacogenetics, advanced cancer, analgesics, palliative care

## Abstract

**Aims:**

To examine the associations between *CYP2D6* and *CYP3A4* polymorphisms, plasma oxycodone and metabolite concentrations, and oxycodone response (dose, pain scores, and adverse effects) in people with pain from advanced cancer.

**Patients & Methods:**

This multi-center prospective cohort study included clinical data, questionnaires (pain and adverse effects), and blood (pharmacokinetics, DNA). Negative binomial regression and logistic regression were used.

**Results:**

Within 33 participants, there were no differences in oxycodone response between CYP2D6 intermediate/poor metabolisers compared to normal metabolisers.

Higher plasma noroxycodone and noroxycodone/oxycodone concentration ratios had higher odds of uncontrolled average pain (OR 2.44 (95%CI 1.00–5.95), *p* = 0.05 and OR 10.48 (95%CI 1.42–77.15), *p* = 0.02, respectively).

**Conclusions:**

There was no observed benefit in *CYP2D6* genotyping in oxycodone response, however monitoring noroxycodone and oxymorphone concentrations warrant further examination.

## 1. Background

Opioids are the cornerstone of cancer pain management [1–4]. Morphine was originally recommended as the opioid of choice, with oxycodone subsequently recommended as a suitable alternative by the European Association for Palliative Care [1]. The use of oxycodone has since risen significantly to surpass morphine [5].

Oxycodone undergoes extensive liver metabolism in which 45% is metabolized to noroxycodone by CYP3A4/5, and 19% is converted into oxymorphone by CYP2D6 [5,6]. Noroxycodone is inactive toward the mu opioid receptor in humans [6], whereas oxymorphone produces analgesia, but its concentration is substantially lower than that of oxycodone, and thus its analgesic effect is likely to be minimal [6]. Both noroxycodone and oxymorphone are subsequently metabolized into noroxymorphone which does not produce analgesia [7].

The pharmacokinetics of oxycodone is influenced by *CYP2D6* genetic polymorphisms [6]. CYP2D6 enzyme function (metaboliser status) is derived using the combination of a person’s inherited alleles [8]. These combinations are assigned into four phenotype categories based on a person’s inherited diplotype, where each allele is assigned a phenotype activity score – poor metabolisers (PM, activity score 0), intermediate metabolisers (IM, activity score between 0 and <1.25), normal (extensive) metabolisers (NM, activity score between 1.25 and 2.25), and ultrarapid metabolizers (UM, activity score > 2.25) [8,9].

*CYP2D6* allele frequency, and thus its metaboliser status (phenotype), differs vastly between biogeographical populations and ethnicities [10], whereby 36% of the global population are considered *CYP2D6* non-normal metabolisers, and higher in certain countries (e.g., >50% in France, Argentina, and Algeria) [10]. *CYP2D6* is the only gene that has pharmacogenomic prescribing guidance for any opioid; however, this is currently limited to the weak opioids codeine and tramadol [11], limiting its application in cancer pain prescribing where more potent opioids are usually required [2,9,12,13]. Interest exists for whether response to oxycodone is associated with the various *CYP2D6* phenotypes. Clinical case studies and those in healthy volunteers show that PMs experienced reduced analgesia/antinociception from oxycodone [14,15], whereas UMs experienced greater analgesia [16,17] and adverse effects [16,18]. However, in postoperative [18,19], breastfeeding [20], and cancer [21] patients there was no influence of PMs or UMs on pain scores and adverse effects. Furthermore, these studies are few, mostly with small sample sizes, and only one study included cancer patients. There is no current recommendation for change in oxycodone dose or prescribing for the different *CYP2D6* phenotypes for cancer-related pain [11,22].

*CYP3A4* genetic polymorphisms have also been found to affect oxycodone pharmacokinetics but have been less studied than *CYP2D6* [6]. The *CYP3A4 × 1B* allele, which increases CYP3A4 enzyme activity [23], shows wide differences in prevalence between populations (66–86% African; 2–4% European, 0% Asian) [6,24] and may result in poorer analgesia due to increased noroxycodone formation [6,23]. The *CYP3A4 × 22* decreased function allele causes reduced CYP3A4 enzyme activity [25] and may result in greater analgesia due to higher oxycodone and potentially oxymorphone concentrations. In contrast to genetic pharmacokinetic factors, current evidence points toward CYP3A4 enzyme activity being influenced more by medications (CYP3A4 inducers or inhibitors) [26] and the proinflammatory mediator IL6 which markedly downregulates mRNA transcription of *CYP3A4* expression [27]. IL6 is most commonly assessed clinically through C-reactive protein (CRP), a marker of inflammation, which IL6 also regulates [27].

Our study aimed to examine whether the predominant pathway for oxycodone metabolism [6] – CYP2D6 and CYP3A4 – metaboliser status affect oxycodone dose, pain scores, and adverse effects in an Australian patient population with pain from advanced cancer, a population which commonly requires oxycodone [28]. We secondarily aimed to examine associations between plasma concentrations of oxycodone and its metabolites with these same clinical outcomes, and their correlations with demographic (age, sex, functional status) and biochemical variables (albumin, C-reactive protein). This will add to the current evidence base by examining correlations between clinical outcomes, *CYP2D6* genotype, and plasma oxycodone and metabolite concentrations.

## 2. Methods

*Design and Population*: Adult inpatients with pain from incurable advanced cancer were recruited into this multi-center prospective cohort study. All participants received stable doses of slow-release oxycodone for pain for at least 3 days before enrollment. Five tertiary metropolitan hospitals with cancer and palliative care services in Australia participated in the study from April 2019 to May 2020. The study was approved by the St Vincent’s Hospital Melbourne Ethics Committee (HREC 252/18). All participants provided written informed consent. The reported study is a substudy of a larger feasibility study, published elsewhere [29]; hence, power calculations were not specifically performed.

*Data Collection*: At study entry, participant demographics, clinical and cancer characteristics (including the Australian-modified Karnofsky Performance Status (AKPS)), oxycodone details (dose, frequency, time between last oxycodone administration and blood sampling), and concurrent medications data were collected to allow for inclusion of CYP2D6 inhibitors and CYP3A4 inhibitors/inducers in the analysis [30]. Pain severity was measured through patient-reported instruments (Brief Pain Inventory-Short Form (BPI-SF), Edmonton Classification System for Cancer Pain (ECS-CP), and Alberta Breakthrough Pain Assessment Tool). Adverse effects were assessed using the Edmonton Symptom Assessment System Revised scale (ESAS-R). All instruments used were validated.

*Pharmacokinetic analyses*: Blood samples were collected just before the administration of the next slow-release dose. Biochemistry (eGFR, C-reactive protein (CRP), and albumin) were collected at the same time. Blood samples for pharmacokinetic analysis were collected into 6 ml lithium heparin tubes and then centrifuged at 1,500 rpm for 5 min. Plasma was aliquoted and stored at −80°C before analysis.

Plasma oxycodone and metabolites (oxymorphone, noroxycodone) were quantified by UHPLC-MS/MS with limits of quantification of 0.3 ng/ml for each analyte [31].

*Genotyping Procedures*: Peripheral blood was collected into a 9 ml EDTA (ethylenediaminetetraacetic acid) tube and frozen at −20°C at time of collection, then later thawed to room temperature for DNA extraction using the QiaAmp DNA Blood Maxi Kit (Qiagen N.V., Venlo, Netherlands). The selection of an appropriate genotyping platform was driven by the nature of *CYP2D6* which is highly polymorphic, subject to copy number variations, structural variants, and pseudogenes, and has rare variants that are not routinely detected [11]; thus, a platform capable of characterizing *CYP2D6* is vital. The Infinium Global Diversity Array with Enhanced PGx (Illumina®, Inc, San Diego, California) was chosen based on its ability to use assay techniques for pseudogene disambiguation to accurately characterize the CYP genes and variants. Phenotypic interpretation and assignment of *CYP2D6* and *CYP3A4* genotypes were performed by Translational Software©,a service which works together with the chosen array platform provider. The phenotypic interpretation results were also checked by the investigators based on current updated Clinical Pharmacogenetics Implementation Consortium and Dutch Pharmacogenetics Working Group guidelines [8,11].

*Pain and Adverse Effect Groupings*: Moderate-severe pain was defined as average pain score ≥4, and uncontrolled breakthrough pain at frequency of ≥4 episodes/day based on acceptable conventions of these definitions [32,33]. Adverse effects were divided into two categories: (1) Opioid adverse effects (at least 2 of: nausea, itch, hallucinations, hiccups, constipation, or drowsiness at ≥4/10), and (2) Poor Psychological Wellbeing (at least 2 of: depression, anxiety, tiredness, or wellbeing scores of ≥4/10).

*Statistical Analyses*: Descriptive results are presented using median (interquartile range (IQR)) and count (frequency). *CYP3A4* genotypes were not compared as no *CYP3A4 × 1B* alleles were found, and there was only one non-NM (*1/*22). CYP2D6 IMs and PMs were grouped together due to low numbers of PMs (n = 2), and then compared to NMs. For participants carrying rare structural variants, the most frequently observed genotype in the published literature was assigned. In particular, the tandem arrangement haplotype *13+*2 (which has one *13 hybrid gene copy upstream of one *2 gene copy on the same allele) was assigned as *2, and *68+*4 (which has one *68 hybrid gene copy upstream of one *4 gene copy on the same allele) was assigned as *4 [34]. Linear regression was used to compare plasma concentrations by CYP2D6 metaboliser status. Due to skewed distribution of the outcomes (plasma concentrations), these were transformed using natural logarithm and results are presented as mean fold difference with 95% confidence intervals. Analysis was adjusted for pre-specified confounders (sex, age, AKPS, eGFR, time since last oxycodone dose, and total oxycodone dose). As there were no CYP2D6 inhibitors nor CYP3A4 inducers/inhibitors [30] taken by the participants, these were not adjusted for in the analysis. Negative binomial regression and logistic regression were used for pain outcomes and adverse effects. Analyses were adjusted for age and time since last oxycodone dose. Pearson’s correlation was used to evaluate the associations between serum concentrations and demographic/biochemical variables. Values of r <0.4 were considered weak correlations, 0.4–0.6 moderate correlations, and >0.6 were considered strong correlations [35]. Analysis was performed using Stata 17 (StataCorp LCC). *p* values of <0.05 were considered statistically significant. Hardy–Weinberg equilibrium exact test was conducted using PLINK2.0 software (χ^2^ test cutoff p ≥ 0.05) [36].

## 3. Results

### 3.1. Participant characteristics

The study comprised 33 inpatients with cancer-related pain. Over half were male (61%, *n* = 20), and median age was 63 years (IQR 40, 60) (Table 1). For CYP3A4, all but one patient (97%; *n* = 32) were NMs (Supplementary Table S1). For *CYP2D6*, the array used was not able to determine genotype for 3 patients (unknown/indeterminate). These are assigned indeterminate by convention for individuals carrying one or two *(star) alleles that have uncertain function [11]. Of the 30 remaining participants, 60% (*n* = 18) were CYP2D6 NMs, 33% (*n* = 10) IMs, and 7% (*n* = 2) PMs; there were no ultrarapid metabolisers. There was no difference in the median time since diagnosis (NM: 17.7 months (IQR 2.7 to 50.4); IM/PM 7 months (IQR 1.2 to 24.3), *p* = 0.42) nor the median duration since the last oxycodone dose (NM: 10 h (IQR 6 to 13); IM/PM 5.8 h (IQR 3.3 to 12.5), *p* = 0.09). The genotype distributions are shown in Supplementary Table S1. Genotype distributions were in Hardy–Weinberg equilibrium (χ^2^ test, *p* ≥ 0.05). None of the participants were prescribed moderate or strong CYP2D6 inhibitors nor CYP3A4 inhibitors or inducers. Median survival was 66 days (IQR 21 to 186).

**Table 1.** Demographics for total sample and according to CYP2D6 metabolizer status.

Characteristic	Total Sample (*n* = 33), Median (IQR)	CYP2D6 Metaboliser Status (*n* = 30)	
		Intermediate/Poor Metaboliser (*n* = 12), Median (IQR)	Normal Metaboliser (*n* = 18), Median (IQR)
Male Sex, n (%)	20 (61%)	7 (58%)	12 (67%)
Age (years)	63 (55, 72)	66 (58.5, 72.5)	63 (49, 70)
Australian Karnofsky Performance Score (AKPS)	50 (40, 60)	60 (50, 65)	50 (40, 60)
BMI (kg/m^2^)	27 1 (24.1, 29.4)	27.0 (24.1, 30.9)	27.4 (23.4, 31.6)
eGFR (mL/min/1.73 m^2^)	90 (73, 90)	90 (73.5, 90)	90 (73, 90)
C-reactive Protein (mg/L)	61.8 (24.2,133.8)	53.0 (24.2, 118.8)	72.9 (30.25, 130.5)
Albumin (g/L)	28 (23, 33)	29.5 (21.5, 33)	29 (24, 34)
Time since diagnosis (months)	8.5 (1.5, 44.8)	7.0 (1.2, 24.3)	17.7 (2.7, 50.4)
Total oxycodone dose (slow + immediate release)	125 (67.5, 195)	115 (71.25, 150)	180 (60, 262.5)
Time since last oxycodone dose (hours)	9.00 (4.50, 12.83)	5.8 (3.3, 12.5)	10.00 (6.00, 13.00)
*Cancer Type*			
Lung	11 (33%)	3 (25%)	8 (44%)
Gastrointestinal	10 (30%)	4 (34%)	4 (23%)
Breast	5 (15%)	2 (17%)	2 (11%)
Prostate	3 (9%)	1 (8%)	2 (11%)
Other (CNS, Urological, Lymphoma)	5 (15%)	2 (16%)	3 (18%)

### 3.2. Pharmacokinetics

**Figure 1.**
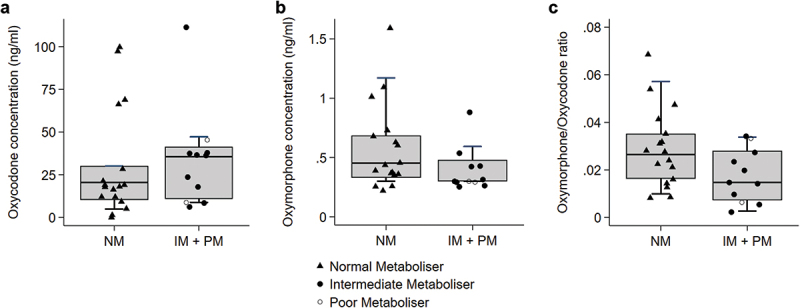
Box and whisker plots of median plasma concentrations (ng/ml) of (a) oxycodone, (b) oxymorphone, and (c) oxymorphone/oxycodone ratio for CYP2D6 normal metabolisers compared to intermediate and Poor metabolisers.

Total oxycodone dose was similar between the two CYP2D6 phenotype groups, (NM 180 mg (IQR 60 to 262.5); IM/PM 115 mg (IQR 71.25 to 150); *p* = 0.29) (Table 1). There was no statistically significant difference in the median plasma oxycodone concentration between the two groups (NM 20.5 ng/ml (IQR 10.4 to 30.1); IM/PM group 35.6 ng/ml (IQR 10.9 to 41.4); *p* = 0.95)) and similarly for plasma oxymorphone (NM 0.43 (IQR 0.31 to 0.69); IM/PM 0.30 (IQR 0.30, 0.48) *p* = 0.24) and oxymorphone/oxycodone ratios (NM 0.03 (IQR 0.02 to 0.04); IM/PM 0.02 (IQR 0.01 to 0.03)); *p* = 0.31) (Table 2, Figure 1). One IM had unquantifiable oxycodone and oxymorphone concentrations (and low noroxycodone concentrations (3.13 ng/ml)) and was excluded from this analysis. After adjusting for potential confounders, the differences between groups remained similar, where the mean fold difference in oxycodone and oxymorphone concentrations, and the oxymorphone/oxycodone metabolic ratio did not reveal statistically significant differences (*p* = 0.31 to 0.95). Plasma noroxycodone concentrations for the entire cohort was a median of 27.6 ng/ml (IQR 13.7 to 92.1).

**Table 2.** Comparisons of plasma oxycodone and oxymorphone concentrations and metabolite ratio for CYP2D6 intermediate and poor metabolisers compared to normal metabolisers.

Plasma Concentration	Intermediate/Poor Metaboliser, Median (IQR)	Normal Metaboliser, Median (IQR)	Mean Fold Difference (95% CI)*	P value
Oxycodone (ng/ml)	35.6 (10.9, 41.4)	20.5 (10.4, 30.1)	1.02 (0.52, 2.00)	0.95
Oxymorphone (ng/ml)	0.30 (0.30, 0.48)	0.43 (0.31, 0.69)	0.75 (0.45, 1.24)	0.24
Oxymorphone/Oxycodone Ratio	0.02 (0.01, 0.03)	0.03 (0.02, 0.0)	0.73 (0.39, 1.36)	0.31

*adjusted for gender, age, AKPS, eGFR, time since last oxycodone dose, and total oxycodone dose.

### 3.3. Pain and adverse effect outcomes according to metaboliser status

Pain outcomes were similar across the two CYP2D6 groups (Table 3), having the same median worst and average pain scores (both 8 and 5, respectively), and with a similar proportion of participants in both groups experiencing uncontrolled breakthrough pain (28% (*n* = 5) NMs vs 25% (*n* = 3) in IM/PMs; OR 0.94 (95% CI 0.33, 17.13), *p* = 0.95). There were no statistically significant differences (*p* > 0.44) in adverse effect outcomes between the IM/PM and NM groups.

**Table 3.** Pain and adverse effect outcomes for CYP2D6 intermediate and poor metabolisers compared to normal metabolisers.

Clinical Outcomes	Intermediate/Poor Metaboliser	Normal Metaboliser	Adjusted analysis*	
			IRR/OR (95% CI)	P value
**Pain Outcomes**				
Worst pain, median (IQR)	8 (7, 9)	8 (6, 10)	1.03 (0.77, 1.38)	0.86
Average pain, median (IQR)	5 (4, 7)	5 (4, 6)	n/a	0.44
Breakthrough pain ≥ 4, n (%)	3 (25%)	5 (28%)	0.94 (0.15, 5.75)	0.95
**Adverse Effect Outcomes**				
Nausea, n (%)	4 (33%)	6 (33%)	1.05 (0.19, 5.71)	0.96
Drowsiness, n (%)	9 (75%)	12 (67%)	2.37 (0.33, 17.13)	0.39
Opioid Adverse Effects, n (%)	7 (58%)	8 (44%)	1.44 (0.26, 7.91)	0.67
Poor Psychological Wellbeing, n (%)	8 (67%)	14 (78%)	0.73 (0.11, 4.94)	0.74

*adjusted for age and time since last oxycodone dose; n/a: model did not converge.

### 3.4. Pain and adverse effect outcomes according to metabolite concentrations and oxycodone dose

Higher plasma noroxycodone and noroxycodone/oxycodone concentration ratios were associated with higher odds of uncontrolled average pain (OR 2.44 (95% CI 1.00 to 5.95), *p* = 0.05 and OR 10.5 (95% CI 1.42,77.15), *p* = 0.02, respectively) (Table 4). Higher total oxycodone dose was significantly associated with higher likelihood of uncontrolled average and breakthrough pain ((OR 4.32 (95%CI 1.10,16.99), *p* = 0.04; and OR 6.4 (95%CI 1.41,29.08) *p* = 0.02, respectively). Although not statistically significant, there was some association between higher oxymorphone concentrations and higher odds of nausea and psychological distress (OR 3.55 (95% CI 0.91,13.92), *p* = 0.07; and OR 3.90 (95% CI 0.94,16.15), *p* = 0.06, respectively).

**Table 4.** Pain and adverse effect outcomes based on oxycodone dose and metabolite concentrations.

Variable	Outcome	N = 33	Metabolite Concentrations			Metabolic Ratios		Total Oxycodone Dose (SR+IR)
			Oxycodone (ng/ml)	Oxymorphone (ng/ml)	Noroxycodone (ng/ml)	Oxymorphone/Oxycodone Ratio	Noroxycodone/Oxycodone Ratio	
Average Pain	Score <4	6	16.19 (12.13, 22.93)	0.34 (0.33, 0.45)	6.86 (5.45, 8.47)	0.03 (0.01, 0.03)	0.45 (0.30, 0.71)	60 (30, 100)
	Score ≥4	26	20.53 (10.21, 40.78)	0.42 (0.30, 0.67)	28.74 (18.44, 80.02)	0.03 (0.01, 0.04)	1.60 (1.24, 2.24)	142.5 (90, 240)
	Odds Ratio* (95%CI)		1.06 [0.39,2.89]	2.5 [0.30,20.42]	2.44 [1.00,5.95]	1.45 [0.38,5.57]	10.48 [1.42,77.15]	4.32 [1.10,16.99]
	p value		0.92	0.40	0.05	0.59	0.02	0.04
Breakthrough Pain	Daily Freq. <4	19	20.53 (12.13, 24.05)	0.453 (0.3, 0.64)	23.10 (8.47, 33.29)	0.03 (0.02, 0.03)	1.46 (.71, 1.96)	100 (45, 135)
	Daily Freq. ≥4	10	19.54 (8.88, 41.37)	0.362 (0.3, 0.619)	35.91 (15.65, 76.21)	0.03 (0.01, 0.04)	2.00 (1.19, 2.48)	217.5 (180, 300)
	Odds Ratio* (95%CI)		1.05 [0.45,2.49]	0.81 [0.20,3.36]	1.50 [0.81,2.78]	0.81 [0.27,2.43]	1.84 [0.72,4.69]	6.4 [1.41,29.08]
	p value		0.91	0.78	0.20	0.71	0.20	0.02
Nausea	Score <4	21	18.12 (10.37, 30.15)	0.333 (0.30, 0.64)	24.81 (13.27, 40.79)	0.03 (0.01, 0.03)	1.46 (1.04, 2.04)	120 (60, 180)
	Score ≥4	11	30.70 (16.19, 41.37)	0.581 (0.34, 0.66)	28.74 (10.90, 80.02)	0.03 (.02, 0.03)	1.51 (0.82, 2.54)	180 (90, 240)
	Odds Ratio* (95%CI)		1.75 [0.78,3.92]	3.55 [0.91,13.92]	1.11 [0.67,1.83]	0.88 [0.31,2.49]	1.08 [0.51,2.33]	1.64 [0.74,3.61]
	p value		0.18	0.07	0.70	0.82	0.83	0.22
Drowsiness	Score <4	10	17.01 (11.90, 19.54)	0.34 (0.33, 0.64)	21.06 (5.45, 76.21)	0.03 (0.02, 0.04)	1.19 (0.71, 1.96)	97.5 (45, 300)
	Score ≥4	22	22.33 (10.66, 39.93)	0.41 (0.30, 0.63)	26.77 (16.81, 68.51)	0.03 (0.01, 0.03)	1.51 (1.09, 2.19)	130 (90, 195)
	Odds Ratio* (95%CI)		1.29 [0.59,2.83]	0.77 [0.25,2.38]	1.50 [0.85,2.66]	0.5 [0.16,1.52]	1.31 [0.58,2.95]	1.26 [0.59,2.70]
	p value		0.53	0.65	0.17	0.22	0.52	0.55
Psychological Distress	Absent	17	16.19 (10.37, 22.93)	0.34 (0.30, 0.48)	22.03 (10.90, 31.13)	0.03 (0.02, 0.03)	1.51 (.87, 2.16)	120 (60, 180)
	Present	11	39.67 (19.54, 65.23)	0.62 (0.30, 0.98)	54.80 (15.65, 115.89)	0.02 (0.01, 0.03)	1.39 (.82, 2.53)	135 (90, 300)
	Odds Ratio* (95%CI)		1.76 [0.79,3.91]	3.90 [0.94,16.15]	1.19 [0.72,1.99]	0.89 [0.31,2.51]	1.16 [0.51,2.63]	1.73 [0.75,4.00]
	p value		0.17	0.06	0.50	0.82	0.72	0.20

*adjusted for age and time since last oxycodone dose.

### 3.5. Correlations between metabolite concentrations and demographic variables

There was a weak positive correlation between age and plasma oxycodone (*r* = 0.34), oxymorphone (*r* = 0.23), and noroxycodone (*r* = 0.31) concentrations and a negative correlation with the plasma oxymorphone/oxycodone concentration ratio (*r* = −0.25) (Table 5). Greater AKPS was moderately correlated with plasma oxycodone concentrations (*r* = 0.49) and inversely correlated with oxymorphone/oxycodone ratio (*r* = −0.41). Noroxycodone/oxycodone ratio was moderately correlated with albumin concentration (*r* = 0.41) but inversely correlated with CRP (*r* = −0.38).

**Table 5.** Associations between oxycodone and metabolite concentrations with demographic and biochemical variables.

Oxycodone and Metabolites Concentration	Age	AKPS	Albumin (g/L)	CRP (mg/L)
Oxycodone	0.34	0.49	−0.11	−0.01
Oxymorphone	0.23	0.26	0.05	−0.14
Oxymorphone/oxycodone ratio	−0.25	−0.41	0.16	−0.08
Noroxycodone	0.31	0.38	0.08	−0.15
Noroxycodone/oxycodone ratio	0.18	0.09	0.41	−0.38

Pearson’s correlation coefficient (r).

## 4. Discussion

### 4.1. Lack of difference in pain and adverse effects outcomes

The relative contribution of plasma oxycodone concentrations compared to oxymorphone in terms of analgesia and adverse effects is controversial. Three studies in healthy volunteers given ≤20 mg oxycodone subjected to cold pressor and electrical stimulation tests show that PMs experience inferior analgesia and less adverse effects, whereas UMs experience superior analgesia, and greater sedation and respiratory depression [16,17]. A sponsored open-label randomized trial comparing oxycodone and oxymorphone oral preparations also showed that the oxymorphone group required only half the dose compared to the oxycodone group to achieve cancer pain control [37]. These studies would support the notion that oxymorphone is responsible for some of the analgesia from oxycodone administration. On the contrary, Babalonis et al. [38] found that oxymorphone did not produce analgesia in response to pressure algometer testing and only did so for cold pressor thresholds at higher doses, whereas oxycodone produced analgesia for both pain tests at all studied dose levels (10 mg, 20 mg, 40 mg). Oxycodone also produced two-fold greater central mu opioid agonist physiological effects (pupil constriction, respiratory depression). Lalovic et al. [7] also observed that the central opioid effects of oxycodone (analgesia, respiratory depression) were from oxycodone alone, attributing the lack of contribution of oxymorphone to analgesia to either substantially lower relative concentrations, as shown in this study, or reduced brain uptake.

In our study, the minor differences in plasma oxycodone and oxymorphone concentrations between NMs and IM/PMs did not translate into any differences in pain outcomes (average, worst, breakthrough pain) nor were there significant differences seen in the adverse effects measured (nausea, drowsiness, opioid adverse effects, psychological wellbeing) between both groups. This is similar to a study by Andreassen et al. comparing CYP2D6 PMs, EMs, and UMs within a large cancer cohort on oxycodone, who found no differences in pain intensity, nausea, tiredness, and cognitive function between these groups [21]. Another similar study comparing NMs and PMs in the post-operative setting also revealed similar expected differences in serum concentrations but no difference in analgesic effect [19]. Our study, like these other clinical studies conducted, suggests that there is no clinically significant difference in pain and adverse effect outcomes in a cancer population based on whether they are CYP2D6 NMs, or IM/PMs.

### 4.2. Noroxycodone associations with pain and markers of cancer cachexia

Higher plasma noroxycodone concentrations were statistically significantly associated with over twice the odds of experiencing moderate-severe pain. One reason could be that the relative clearance of oxycodone to noroxycodone is much greater than normal such that the relative clearance to oxymorphone is markedly reduced leading to the systemic exposure of oxycodone and oxymorphone to be much lower than normal [39]. Noroxycodone has also been found to induce neuroexcitatory effects (allodynia, tremor, and myoclonic jerks) in rodents and may be a potential contributing mechanism [40]. In our study, all patients except one were CYP3A4 NMs, no participants were taking CYP3A4 inducers/inhibitors, and liver and renal function were generally unremarkable; hence, these are not contributors to differences in variability in noroxycodone concentrations. However, our participant population with advanced incurable cancer had a median survival of only 66 days, and it is likely that cancer cachexia was prevalent.

Cancer cachexia is a multifactorial syndrome causing progressive skeletal muscle loss leading to progressive functional deterioration [41] and is associated with increased production of CRP, reduced albumin, and a worse performance status (lower AKPS) [5,39]. Naito et al. [39,42] found that people with cancer cachexia, as measured by high CRP and low albumin, experience a reduction in CYP3A activity but not CYP2D6 activity. This would cause lower noroxycodone concentrations and thus higher oxycodone and oxymorphone concentrations by a greater contribution of the CYP2D6 pathway to oxycodone metabolism [5,39,42]. Similar to Naito’s study, we found that noroxycodone metabolic ratio was proportionally correlated with albumin concentration, and inversely correlated with CRP. Additionally, the clinical and biochemical markers of cachexia (hypoalbuminaemia, high CRP, worse performance status) were each correlated with lower noroxycodone concentrations or its metabolic ratio. It is plausible that downregulation of CYP3A activity may cause lower oxycodone dose requirements in those with cancer cachexia, and although this has not been studied, confirming this in a clinical setting may help clinicians toward cautious dosing of oxycodone in patients with cancer cachexia.

### 4.3. Association between oxymorphone concentrations and adverse effects

Most pharmacokinetic studies examining adverse effects of oxycodone report on its central effects relating to mu-opioid receptor binding, such as sedation and respiratory depression due to ease of objective measurements. However, nausea is the second commonest adverse effect from oxycodone after constipation [43] and is experienced by 40% of cancer patients who are prescribed opioids [44]. One study found that nausea was the most feared adverse effect and that patients would rather accept a higher pain level to avoid this side effect [45]. The mechanism of oxycodone-induced nausea is likely multifactorial, and a combination of mu-opioid receptor agonism causing reduced gastrointestinal motility, increased vestibular sensitivity, and stimulation of the chemoreceptor trigger zone [46]. Similarly, opioid use is associated with reduced mood and psychological distress through a variety of mechanisms such as reduction in cortisol levels, serotonergic dysregulation, and epigenetic misregulation of the endogenous opioid pathway [47]. No study has previously examined whether oxycodone or its metabolites are specifically associated with nausea or psychological distress. In our relatively small sample, we found that those with higher plasma oxymorphone concentrations showed some relationship with increased likelihood of nausea (OR ~ 3.5, *p* < 0.1) and psychological distress (OR ~ 4, *p* < 0.1), which may warrant follow-up in larger numbers of patients.

### 4.4. Age and oxycodone

We found that greater age was correlated with higher oxycodone concentration and a lower oxymorphone to oxycodone metabolic ratio, which is consistent with reduced clearance and prolonged half-life of oxycodone with age [5,6]. Additionally, age was also proportional to greater noroxycodone concentrations, which are seen in elderly patients in other studies [6]. Authors of previous studies have called for oxycodone dose reductions of up to 50% in elderly patients and a greater interval between doses [5]. Our finding of age correlations with oxycodone and its metabolites further emphasize the importance of cautious dosing in the elderly.

### 4.5. Limitations and future directions

There are several limitations to this study. First, the unavailability of UMs and small number of PMs limited the comparisons that could be made. The prevalence of UMs and PMs are low (2.8% and 5.7% respectively) [10], and a larger sample size powered to the respective *CYP2D6* and *CYP3A4* allele frequency and metaboliser status distributions may reveal more meaningful or definitive results. Second, our sample was heterogenous in terms of cancer types which could have different pain syndromes and severity, although major differences in opioid dosing are unlikely [48] and our sample represents what is seen in clinical practice. Although patients are assigned an indeterminate CYP2D6 phenotype by convention due to their inheritance of *(star) alleles with uncertain function, this may perpetuate the uncertainty relating to those who inherit these alleles in both research and clinical practice.

The relationship between greater noroxycodone concentrations causing greater odds of experiencing moderate-severe pain deserves further investigation. There were interesting potential associations between oxymorphone and adverse effects (nausea, psychological distress) that could be worthy of follow-up in future studies. In addition to low albumin and high CRP, low AKPS is a plausible additional surrogate of cachexia, and this relationship to noroxycodone could also be studied in future.

## 5. Conclusion

Our study adds to the limited evidence in the combined assessment of CYP2D6 metaboliser status, oxycodone serum concentration and its metabolites, with clinical phenotype outcomes. CYP2D6 metabolizer status was not associated with oxycodone dose nor pain (average, worst, breakthrough) or adverse effect outcomes in IM/PMs compared to NMs who have advanced cancer.

The monitoring of oxycodone metabolites (noroxycodone and oxymorphone) warrant future investigation into their relationships with pain and adverse effect outcomes accounting for the presence of cachexia and a proinflammatory state, and also other potential genetic contributors to variability in pharmacokinetic outcomes.

## Supplementary Material

Supplemental Material

## Data Availability

The data presented in this study are available on request from the corresponding author. The data are not publicly available due to privacy reasons.

## Article highlights

**Background**

- Oxycodone pharmacokinetics is influenced by *CYP2D6* and *CYP3A4* genetic polymorphisms.
- Most studies examining their associations with clinical outcomes are in non-cancer settings and demonstrate inconsistent results.**Methods**
- This multi-center prospective cohort study recruited patients receiving oxycodone for pain due to advanced cancer.
- Blood samples were collected for DNA and pharmacokinetic analysis. Plasma oxycodone and metabolites (oxymorphone, noroxycodone) were quantified by UHPLC-MS/MS.**Results**
- There were no differences in pain (average, worst, breakthrough) and adverse effect outcomes (*p* > 0.44) between CYP2D6 intermediate/poor metabolisers compared to normal metabolisers. Higher plasma oxymorphone concentrations showed a weak relationship with increased likelihood of nausea and psychological distress (both *p* < 0.1).
- Higher plasma noroxycodone and noroxycodone/oxycodone concentration ratios were associated with higher odds of uncontrolled average pain (OR 2.44 (95%CI 1.00 to 5.95), *p* = 0.05 and OR 10.48 (95%CI 1.42 to 77.15), *p* = 0.02, respectively).**Discussion & Conclusions**
- CYP2D6 metaboliser status was not associated with oxycodone dose, pain (average, worst, breakthrough), or adverse effect outcomes in CYP2D6 intermediate/poor metabolisers compared to normal metabolisers in this small study.
- Monitoring of noroxycodone and oxymorphone concentrations warrants follow-up in larger numbers of patients accounting for pro-inflammatory states and other potential genetic contributors.
