# Supplementary material for: Do CYP2D6 genotypes affect oxycodone dose, pharmacokinetics, pain, and adverse effects in cancer?
Source: Pharmacogenomics. 2024 Dec 4;25(14-15):579–86. doi: 10.1080/14622416.2024.2430161 (PMC11703413; doi:10.1080/14622416.2024.2430161)
Supplement: Supplemental Material [file IPGS_A_2430161_SM9182.docx]

**Supplementary Table 1: Distribution of *CYP2D6* and *CYP3A4* genotypes**

| ***CYP2D6* Genotype** | **N=30^α^** | **% (total sample)** |
| --- | --- | --- |
| *Normal Metaboliser* | *18* | *60%* |
| *1/*1 | 4 | 13% |
| *1/*2 | 9 | 30% |
| *1/*9 | 1 | 3% |
| *10/*2 | 1 | 3% |
| *2/*2 | 3 | 10% |
| *Intermediate Metaboliser* | *10* | *33%* |
| *1/*4 | 5 | 17% |
| *1/*5 | 2 | 7% |
| *2/*4 | 2 | 6% |
| *2*4N | 1 | 3% |
| *Poor Metaboliser* | *2* | *7%* |
| *4/*4 | 2 | 7% |
| ***CYP3A4* Genotype** | **N=33** | **% (total sample)** |
| *Normal Metaboliser* | 32 | 97% |
| *1/*1 | 32 | 97% |
| *Intermediate Metaboliser* | 1 | 3% |
| *1/*22 | 1 | 3% |

**^α^**excluding 3 participants with unknown genotype
